# Coffee Wastes: A Sustainable Source of Natural Compounds Suppressing Colorectal Cancer Cell Viability

**DOI:** 10.1155/omcl/8034350

**Published:** 2025-12-28

**Authors:** Mariavittoria Verrillo, Paola Cuomo, Cristina Pagano, Fabrizio Martora, Riccardo Spaccini, Rosanna Capparelli, Salvatore Velotto

**Affiliations:** ^1^ Department of Agricultural Sciences, University of Naples “Federico II”, Portici, Italy, unina.it; ^2^ Interdepartmental Research Centre on Nuclear Magnetic Resonance (NMR) for the Environment, Agroo-Food and New Materials (CERMANU), University of Naples “Federico II”, Portici, Italy, unina.it; ^3^ Department of Molecular Medicine and Medical Biotechnology, University of Naples “Federico II”, Naples, Italy, unina.it; ^4^ Dermatology Unit, Department of Clinical Medicine and Surgery, University of Naples “Federico II”, Naples, Italy, unina.it; ^5^ Task Force on Microbiome Studies, University of Naples Federico II, Naples, Italy, unina.it; ^6^ Department of Human Science and Quality of Life Promotion, San Raffaele University, Rome, Italy, unisr.it

**Keywords:** agri-food wastes, cell apoptosis, colorectal cancer, humic substances, natural antioxidant supplements

## Abstract

Colorectal cancer (CRC) is one of the leading causes of cancer‐related deaths worldwide. Emerging evidence suggests a rising incidence of CRC in younger adults, emphasizing the urgent need for innovative therapeutic strategies. The increasing attention on circular economy approaches has heightened interest in discovering natural compounds derived from recycled agri‐food waste. These compounds are particularly promising due to their large array of bioactive functional components. In this study, we investigated the efficacy of a green compost derived from coffee wastes, known as humic substance (HS), in reducing CRC cell viability. Chemical characterization of HS from composted coffee waste (HS‐COF) using ^13^C Cross‐Polarization Magic‐Angle Spinning Nuclear Magnetic Resonance (CPMAS NMR) spectroscopy and offline pyrolysis Tetramethylammonium Hydroxide‐Gas Cromatography Mass‐Spectrometry (TMAH‐GC–MS) revealed an abundance of phenolic compounds derived from lignin residues. Specifically, chlorogenic acid (ClA) was identified as the major component and primary agent responsible for the biological effects of HS‐COF. Our *in vitro* results demonstrated that HS‐COF selectively inhibits HT‐29 cell viability, migration, and proliferation by inducing programed cell death through the activation of the tumor necrosis factor‐*α* (TNF‐*α*) signaling pathway and disruption of calcium homeostasis. Additionally, HS‐COF exhibited a significant antioxidant activity, indicating its potential to combine a cytotoxic profile with a safety profile, thereby minimizing adverse effects on healthy cells. In conclusion, this study proposes HS‐COF as a valuable adjuvant in CRC therapy, paving the way for its application in the pharmaceutical industry.

## 1. Introduction

Colorectal cancer (CRC) is one of the major causes of cancer‐related deaths worldwide [1]. Although CRC primarily affects older adults (over 65 years), recent evidence shows a rapid increase in cases among people under the age of 50 [2, 3]. This alarming trend contributes to the high mortality rate, as younger people are not routinely screened for CRC and often ignore early symptoms. As a result, CRC in younger patients is frequently diagnosed at an advanced stage, reducing the effectiveness of standard treatments. Therefore, novel and effective therapeutic interventions are needed to prevent or treat this global health challenge.

Emerging studies clearly report the significant contribution of diet and nutritional supplements in cancer therapy [4]. Among these, antioxidants are widely recognized as anticancer adjuvants. They reduce gut inflammation and oxidative stress while promoting cell apoptosis [5, 6]. However, while antioxidants can tackle the tumor progression, they may also inhibit the efficacy of radiotherapy and chemotherapy agents, which exert their antitumoral effect through reactive oxygen species (ROS) production [7]. Of note, these adverse effects have been attributed to an excessive consumption of antioxidant supplements, while no detrimental effects have been reported for the assimilation of antioxidants from food [8].

Coffee is one of the main dietary sources of antioxidant bioactive compounds [9], including melanoidins and caffeic and chlorogenic phenolic acids, which exhibit important antiinflammatory, antioxidant, and anticancer properties [10, 11]. In particular, chlorogenic acid (ClA) was found to induce CRC cell apoptosis through the inhibition of cell cycle via modulation of the p21–p53 signaling, priming the expression of *Caspase 3* and *9* genes, and decreasing MAPK/ERK phosphorylation [12]. Furthermore, acting as an antiinflammatory agent, ClA prevents the nuclear factor kappa‐light‐chain‐enhancer of activated B cells (NF‐kB) pathway, thus modulating the release of tumor necrosis factor‐*α* (TNF‐*α*) [13], which orchestrates different cellular processes, comprising cell survival, proliferation, and death [14].

TNF‐*α* is one of the major pro‐inflammatory cytokines, extensively recognized as a key regulator of inflammaton‐induced carcinogenesis. In opposition, TNF‐*α* can also contribute to tumor suppression by functioning as an apoptosis‐promoting protein. This dual role is mediated by the activation of two distinct signaling routes: (i) the NF‐kB pathway, leading to the transcription of pro‐inflammatory genes which promote cell survival, proliferation, and inflammation [13, 14], and (ii) the c‐Jun N‐terminal kinase (JNK) pathway, which links the expression of pro‐apoptotic genes with cell death [14]. As a result, the cellular outcome depends on the balance between the NF‐kB and JNK reaction sequences, highlighting the critical role of TNF‐*α* in tumorigenesis and its potential therapeutic properties in cancer disease.

In this study, we investigated whether a coffee‐derived humic substance (HS‐COF)—obtained by composting coffee wastes, including immature and defective beans, coffee husks, pulp, coffee silver skin, and spent coffee grounds—could exert anticancer effects by targeting the TNF‐*α* cytokine. ^13^C Cross‐Polarization Magic‐Angle Spinning Nuclear Magnetic Resonance (CPMAS NMR) spectroscopy and Offline pyrolysis Tetramethylammonium Hydroxide‐Gas Cromatography Mass‐Spectrometry (TMAH‐GC–MS) revealed an abundance of phenolic compounds derived from lignin residues and, specifically, of ClA, an intermediate of the lignin biosynthetic process [15]. We found out that HS‐COF significantly inhibits human colon cancer cells. It suppressed HT‐29 cell migration and proliferation by promoting cell death in response to TNF‐*α* increase and calcium homeostasis imbalance. Interestingly, HS‐COF selectively counteracted CRC cells and exhibited a considerable antioxidant activity, highlighting its effectiveness as a potential adjuvant in CRC therapy. Remarkably, in a scenario focused on economic and environmental sustainability, the pharmaceutical application of HS‐COF is a prospective strategy that combines the use of eco‐friendly natural products with the valorization of cost‐effective, widely available agri‐waste sources.

In conclusion, our study proposes HS‐COF as a biocompatible and efficient dietary supplement for CRC treatment in combination with conventional antitumoral drugs.

## 2. Materials and Methods

### 2.1. Green Compost Production and Humic Substances Extraction

Coffee wastes from Kimbo S.R.L. were collected at the Experimental Farm of the University of Naples “Federico II” in Castel Volturno (CE). They were mixed with corn straw and wood chips in a 70/30 (w/w) ratio and processed via aerobic composting for 60 days [16]. The final product was stored in static piles over perforated tubes to maintain optimal aerobic microbial activity. The entire composting process spanned 100 days and consisted of three distinct phases: thermophilic, mesophilic, and maturation. After composting, the green compost was sieved through a 2 mm mesh to isolate organic products called HSs. To extract HS, the compost was treated with 0.1M KOH, shaken for 24 h, centrifuged at 700 rpm for 25 min, and filtered through glass‐wool. The extracted solution was then acidified with 6M HCl to reach a pH of 7.4. Finally, the sample was dialyzed using 1 kDa Spectra/Por membranes and freeze‐dried.

### 2.2. Molecular Characterization of HS (Elemental Content, NMR Spectroscopy, and Off‐Line Thermochemolysis)

To investigate the elemental content (C, H, and N) of HS, the elemental analysis was performed using a UNICUBE analyzer. Infrared spectra were collected with a Perkin Elmer 1720‐X FT‐IR spectrometer (DRIFT accessory) using 12 scans at 6 cm^−1^ resolution after mixing the sample with KBr in an agate mortar.

Solid‐state ^13^C NMR spectra (^13^C‐CPMAS–NMR) were recorded on a Bruker AV‐300 endowed with a 4 mm wide‐bore MAS at 10 kHz spinning frequency, 2 s repetition time, 1 ms contact time, and 30 ms acquisition time over 4000 scans. Spectra were divided into six regions (0–190 ppm) corresponding to specific functional groups: 0–45 ppm (aliphatic‐C), 45–60 ppm (methoxyl‐C and N‐alkyl‐C), 60–110 ppm (O‐alkyl‐C), 110–145 ppm (aromatic‐C), 145–160 ppm (O‐aryl‐C), and 160–190 ppm (carboxyl‐C), and relative spectral areas were used to quantify them. Furthermore, four dimensionless indexes were calculated according to the following equations: O‐alkyl ratio (A/OA) = [(0–45)/(60–110)]; aromaticity index (ARM) = [(110–160)/(0–190)]; hydrophobic index (HB) = ∑[(0–45) + (45–60)/2 + (110–160)]/∑[(45–60)/2 + (60–110) + (160–190)] and lignin ratio (LigR) = [(45–60)/(140–160)].

Whereas the HB, A/OA, and ARM indexes describe biochemical stability and bioactivity of the organic extract, LigR is critical for discriminating signals corresponding to lignin or other phenolic contents [16–18].

For the off‐line thermochemolysis, 500 mg of dried HSs were placed in a quartz boat and treated with 1 mL tetramethyl‐ammonium hydroxide (TMAH, 25% methanol) and pyrolyzed in a tubular furnace (Barnstead Thermolyne), as previously detailed [16, 19]. Released compounds were analyzed via gas cromatography‐mass spectrometry (GC‐MS) by the PE Autosystem XL coupled with a PE Turbomass‐Gold quadrupole [16, 19], and the analysis was performed using the NIST library database, published spectra, and standards.

### 2.3. Determination of Caffeic Acid and Chlorogenic Acid Content

Phenolic fractions of HS were extracted following the protocol described by Martuscielli et al. [20], with some modifications. Different solvent mixtures were used: deionized water and methanolic aqueous solutions at two methanol concentrations 60:40 and 70:30 (v/v). The phenolic analysis was achieved on the 70% methanolic extract. Standard solutions of caffeic acid and CIA (Fluka, Buchs, CH, Switzerland), spanning from 1 to 200 mM, were used for external calibration via UV/Vis spectrophotometry.

### 2.4. Total Phenolic Content and Antioxidant Properties

The Folin–Ciocalteu assay was performed to determine the total phenolic content (TPC) of HS. Briefly, 4 mg of the sample was dissolved using 2 mL methanol/water solution. The obtained solution was mixed with 50 μL of Milli‐Q water and 12.5 μL of Folin–Ciocalteu and then incubated for 90 min. After incubation, absorbance was measured at 760 nm using a Perkin Elmer Lambda 25 UV/Vis spectrometer. Results were expressed as millimoles of gallic acid equivalents (GAEs) per mg of dry extract. Antioxidant activity of compost derivatives was evaluated through ABTS and DPPH assays, as reported by Verrillo et al. [16]. Results were expressed as Trolox equivalent antioxidant capacity (TEAC) based on a Trolox standard calibration curve (*R* ^2^ = 0.997).

### 2.5. Cell Culture

The human colorectal and pancreatic carcinoma cell lines [HT‐29 (RRID: CVCL_0320) and PANC‐1 (RRID: CVCL_0480), respectively] and the human colorectal non‐cancer cell line [CCD‐18Co (RRID: CVCL_2379)] were obtained from the American Type Culture Collection (ATCC, Manassas, VA, USA, #HTB‐38; #CRL‐1469; #CRL‐1459, respectively). All cells were grown in Dulbecco’s modification of Eagle’s medium, high glucose (DMEM; Microtech, Naples, Italy) supplemented with 10% fetal bovine serum (FBS; Microtech, Naples, Italy), 1% penicillin/streptomycin (Microtech, Naples, Italy) and 1% L‐glutamine (Microtech, Naples, Italy) and maintained in a humidified environment at 37°C and 5% CO_2_. All cell lines were authenticated using short tandem repeat (STR) profiling within the last 3 years. All experiments were performed with mycoplasma‐free cells.

### 2.6. HS‐COF and ClA: Cytotoxicity Assay

The cytotoxic effect of (1) HS‐COF on both cancerous and non‐cancerous cells, and (2) ClA on HT‐29 cells was determined by performing the MTT (3‐(4, 5‐dimethylthiazolyl‐2)‐2, 5‐diphenyltetrazolium bromide) assay. Cells were seeded in a 96‐well plate at a density of 0.6 × 10^4^ cells/well and incubated at 37°C in a 5% CO_2_ atmosphere for 24 h. The next day, cells were treated with different concentrations of HS‐COF (spanning from 1 to 500 μg/mL) or ClA (spanning from 50 to 1000 μM) and incubated for a further 24 and 72 h. Untreated cells were used as a negative control. After incubation, cells were washed and further incubated with 20 μL of MTT solution (5 mg/mL; Merck) and 180 μL of cell culture medium/well for 3 h. Finally, the medium was removed, and the resultant formazan crystals dissolved in 200 μL of dimethyl sulfoxide (DMSO). Cellular MTT reductase activity was determined by measuring the absorbance of DMSO extracts at 570 nm, using a microplate reader (NB‐12‐0035; Neo Biotech, Nanterre, France). Results are expressed as the percentage of MTT‐reducing activity of treated *vs* untreated cells, according to the following formula: % cell viability = [(mean absorbance of the sample) – (mean absorbance of the blank)]/[(mean absorbance of the control) – (mean absorbance of the blank)] × 100. IC_50_ was calculated for each cancer cell line using the above‐reported formula and plotting a linear regression curve.

### 2.7. Cell Recovery Assay

The capability of the HT‐29 cells to reverse the HS‐COF‐induced cytotoxicity was determined by performing the MTT (3‐(4,5‐dimethylthiazolyl‐2)‐2,5‐diphenyltetrazolium bromide) assay. HT‐29 cells were seeded in a 96‐well plate at a density of 0.6 × 10^4^ cells/well and incubated at 37°C in a 5% CO_2_ atmosphere for 24 h. The next day, cells were treated with three concentrations of HS‐COF (400, 100, and 25 μg/mL) and incubated for 24 h. After incubation, cells were washed to eliminate the treatment and further incubated with fresh medium for 24 h. Finally, the MTT assay was performed as detailed in the previous paragraph.

### 2.8. Cell Migration Assay

To investigate whether HS‐COF could inhibit the HT‐29 cell migration, an *in vitro* scratch assay was performed, as described by Cuomo et al. [21]. In detail, cells were seeded at a high density in a 12‐well plate and incubated at 37°C in a 5% CO_2_ atmosphere. The next day, cells were treated with mitomycin for 2 h (2 μg/mL) to inhibit cell growth. Cell monolayers were then scratched using a 200‐pipette tip, and a gap was created. After scratching, cells were washed with phosphate‐buffered saline (PBS; Microtech, Naples, Italy) to remove cell debris and further incubated with HS‐COF (400 μg/mL) or ClA (200 μM) for 48 h. Cells were checked under a phase‐inverted microscope, and images acquired before and after 24 and 48 h of HS‐COF or ClA treatment, and finally, analyzed using the ImageJ software. Results are expressed as a percentage of wound closure, according to the following formula: % wound closure = [(wound area_*T* = 0_ – wound area_*T* = 24 or 48_)/(wound area_*T* = 0_)] × 100.

### 2.9. Colony Forming Assay

The effectiveness of HS‐COF as an antitumoral drug was evaluated by assessing its ability to inhibit HT‐29 cell clonal expansion using a clonogenic assay. For this experiment, HT‐29 cells were seeded at a density of 200 cells per well in a 6‐well plate and incubated at 37°C in a 5% CO_2_ atmosphere. After cell attachment, the cells were treated with HS‐COF (400 μg/mL) or ClA (200 μM) and incubated for an additional 15 days under the same conditions. The culture medium was refreshed every 3 days. On the 15th day, the medium was removed, and the cells were gently washed with PBS before being fixed in 400 μL of methanol for 20 min. The methanol was then thoroughly washed off with water, and the cells were stained with 0.5% crystal violet. Colonies consisting of at least 50 cells were counted.

### 2.10. ABTS Assay on Cell Culture

The antioxidant activity of both HS‐COF and ClA was tested on HT‐29 cell lysate according to the method of Pellegrini et al. [22], with some modifications. Cells were seeded at a density of 1 × 10^6^ cells/well in a 6‐well plate and incubated at 37°C in a 5% CO_2_ atmosphere. The next day, cells were washed with PBS and treated with HS‐COF (400 μg/mL) or ClA (200 μM) for 24 and 72 h. After treatments, cells were scraped and centrifuged at 1000 g for 5 min to obtain cell pellets, which were resuspended to 1 mL cold PBS and sonicated on ice. Cell lysates were then centrifuged at 10,000 g for 15 min at 4°C, and supernatants were collected and stored at −80°C until the analysis was performed.

For the analysis, the ABTS solution was diluted with ethanol (0.2% of water) at room temperature, and 10 μL of the obtained mixture was added to 1 mL of the above‐collected supernatants. Absorbance was measured after 2 min using a spectrophotometer (Perkin Elmer Lambda 25 UV/Vis spectrometer) at a wavelength of 734 nm.

### 2.11. Calcium Mobilization Assay

To determine the intracellular calcium concentration of HT‐29 cells following HS‐COF or ClA treatment, a colorimetric assay was performed on cell lysates prepared as reported above. Briefly, cells were seeded at a density of 1 × 10^6^ cells/well in a 6‐well plate and incubated at 37°C in a 5% CO_2_ atmosphere. The next day, cells were washed with PBS and treated with HS‐COF (400 μg/mL) or ClA (200 μM) for 24 h. After treatments, cells were scraped and centrifuged at 1000 g for 5 min to obtain cell pellets, which were resuspended to 1 mL cold PBS and sonicated on ice. Cell lysates were then centrifuged at 10,000 g for 15 min at 4°C, and supernatants were collected and stored at −80°C until the analysis was performed. The analysis was achieved using the calcium colorimetric assay kit (Merck, Missouri, USA; #MAK022) according to the manufacturer’s instructions.

### 2.12. RNA Extraction and Quantitative Gene Expression

Expression levels of *TNF-α*, *FAS*, and T*p53* genes were measured by qRT‐PCR, following the methodology described by Verrillo et al. [19]. HT‐29 cells were seeded at a density of 1 × 10^6^ cells per well in a 6‐well plate and incubated at 37°C in a 5% CO_2_ atmosphere overnight to allow cell attachment. The next day, cells were treated with HS‐COF (400 µg/mL) or ClA (200 µM) for 24 h. After treatment, RNA was extracted using TRIzol reagent (Thermo Fisher Scientific), according to the manufacturer’s instructions. The quality and quantity of RNA were assessed using a NanoDrop 2000c spectrophotometer (Thermo Fisher Scientific). Following RNA purification and genomic DNA digestion with the DNase I, Amplification Grade kit (Invitrogen; #18068015), cDNA was synthesized using the high‐capacity cDNA Reverse Transcription kit (Thermo Fisher Scientific). Real‐time PCR reactions were conducted on a StepOne Real‐Time PCR System (Thermo Fisher Scientific), employing Power SYBR Green PCR Master Mix (Applied Biosystems) as the amplification system. Gene‐specific primers are listed in Table S1. The relative changes in target gene expression were determined using the 2^−*ΔΔ*Ct^ method. GAPDH was used as the housekeeping gene to normalize the data, ensuring accurate quantification of *TNF-α*, *FAS*, and *Tp53* gene expression levels.

### 2.13. Cell Death

The ability of HS‐COF to induce apoptosis in HT‐29 cells was assessed by a DNA ladder assay and quantification of Caspase‐3 and ‐9 (CASP3, CASP9). HT‐29 cells (1 × 10^6^/well) were seeded in 6‐well plates and incubated overnight at 37°C with 5% CO_2_. The next day, cells were treated with HS‐COF (400 µg/mL) or ClA (200 µM) for 24 h. Untreated cells served as a negative control, while 5% DMSO‐treated cells were used as a positive control. For the DNA ladder assay, cell supernatants were collected and centrifuged at 1000 g for 5 min at 4°C. The liquid phase was collected, further centrifuged, and used for caspases quantification, while the resulting pellets, together with the adherent cells, were lysed with 0.1 M NaOH and incubated for 5 min at room temperature. Following incubation, the cell lysate was neutralized with 0.1 M HCl, and the solution was transferred into a fresh tube containing proteinase K at a final concentration of 200 µg/mL and, finally, incubated at 56°C for 1 h to allow protein digestion. DNA was then extracted using the phenol‐chloroform method, and DNA fragmentation was evaluated by agarose gel electrophoresis, following the protocol described by Matassov et al. [23].

Caspase levels were measured in cell supernatants, obtained as reported above, using human CASP3 and CASP9 ELISA Kits (Elabscience; #E‐EL‐H0017 and #E‐EL‐H0663), according to the manufacturer’s instructions.

### 2.14. Statistical Analysis

GraphPad Prism 9.0 (San Diego, CA, USA) was used for statistical analysis. Multiple comparisons, among more than two experimental groups, were performed using one‐or two‐way analysis of variance (ANOVA) followed by Tukey or Bonferroni post‐hoc correction. Data were considered statistically significant when *p*‐value was <0.05.

## 3. Results

### 3.1. Chemical Characterization of HS‐COF

#### 3.1.1. Elemental Composition

The elemental composition of HS‐COF revealed a significant content of both organic carbon and total nitrogen (Table 1) with an estimated organic material (OM) content close to 86%. Compared to most of the humic materials, HS‐COF showed a lower C/N ratio, due to the abundance of N moieties, and a higher H/C ratio, which suggest a predominance of aromatic compounds over aliphatic components [24].

**Table 1:** Elemental composition of humic substances (HS) extracted from coffee grounds (COF) composted residues.

Sample	C (%)	N (%)	H (%)	C/N	H/C
HS‐COF	51.08 ± 0.03	7.6 ± 0.06	4.8 ± 0.01	7.84	1.11

*Note*: Coefficient of variation was always lower than 1%.

#### 3.1.2. Infrared Spectroscopy

**Figure 1:**
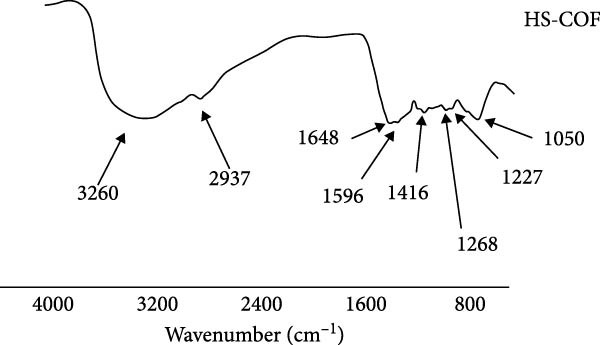
FTIR‐DRIFT spectra of humic substances (HS) extracted from coffee grounds (COF) composted residues.

The DRIFT spectra of HS (Figure 1) displayed characteristic functional groups typical of humic materials derived from green composts [19, 25]. The broad band at 3000–3500 cm^−1^ corresponds to overlapping OH stretching vibrations in alcohols, phenolic and carboxylic acids [18], while the shoulder at 2937 cm^−1^ is assigned to symmetric and asymmetric C–H stretching in methyl and methylene groups of aliphatic structures (Figure 1). Signals at 1648 and 1596 cm^−1^ likely originate from the amide I and II bonds in peptides, with potential contributions from C = O stretching of carboxylate salts and aromatic ring vibrations around 1650 and 1520 cm^−1^, respectively [18, 26]. Peaks at 1416 and 1268 cm^−1^ indicate carboxylic acid rotational vibrations, while the 1220 cm^−1^ peak corresponds to C–O–H in phenolic structures. Finally, the absorbance at 1050 cm^−1^ corresponds to alkyl C–O–H bonds in carbohydrates and polysaccharides (Figure 1).

#### 3.1.3. ^13^C‐CPMAS–NMR Spectroscopy

**Figure 2:**
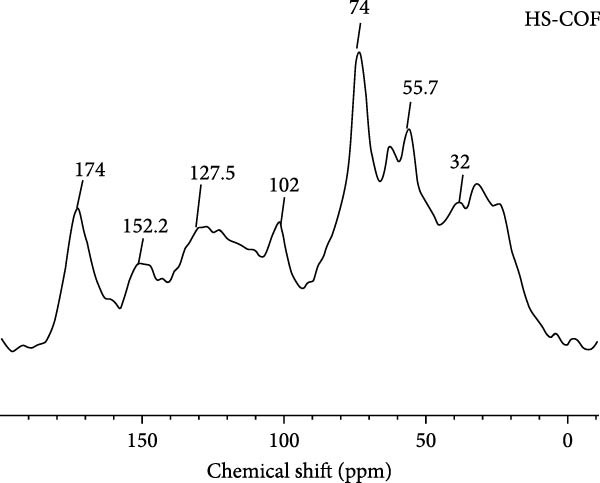
^13^C CPMAS NMR spectra of humic substances (HS) extracted from coffee grounds (COF) composted residues.

The heterogenous composition of humic extracts was confirmed by solid‐state ^13^C‐CPMAS‐NMR spectra (Figure 2).

The carbon distribution emphasized the abundance of O‐alkyl carbons (60–110 ppm) and aromatic carbons (110–160 ppm) from lignocellulose fractions [17], accounting for 53% of the relative area (Table 2).

**Table 2:** Relative abundance percentage of chemical shift intervals (ppm) of ^13^C‐CPMAS–NMR spectra of humic substances (HS) extracted from coffee grounds (COF) composted residues.

Sample	190–160	160–145	145–110	110–60	60–45	45–0	HB/HI^a^	A/AO^b^	LigR^c^	ARM^d^
HS‐COF	12.7	5.4	16.9	30.4	11.7	23.8	1.1	0.8	2.2	0.4

^a^Hydrophobic index (HB): [(0–45) + (45–60)/2 + (110–160)]/[(45–60)/2 + (60–110) + (160–190)].

^b^Alkyl ratio (A/AO): [(0–45)/(60–110)].

^c^Lignin ratio (LigR): [(45–60) 145–160)].

^d^Aromatic index (ARM) = [(110–160)/(0–190)].

NMR resonances in the 0–45 ppm range correspond to methylene and methyl moieties in alkyl chains of lipids from plant waxes, polyesters, and microbial by‐products. The sharp peak at 55 ppm (45–60 ppm range) denotes an overlap of methoxyl carbons, from guaiacyl and syringyl aromatic rings of lignin fragments, and C*α*‐N bonds in oligo‐ and polypeptides [26]. Strong signals in the O‐alkyl region (60–110 ppm) reflect pyranose and furanose structures in mono‐, oligo‐, and polysaccharides (Figure 2). The less intense shoulder at 60 ppm represents out‐of‐plane C6 nuclei, while the broad band at 72 ppm corresponds to coalesced C2, C3, and C5 carbons. Additionally, the 102 ppm peak is attributed to di‐O‐alkyl anomeric carbons [17]. The absence of C4 signals (80–90 ppm) typical of *β*‐1→4 glycosidic bonds and the shift of anomeric carbons to lower chemical shifts suggest the inclusion of depolymerized lignocellulose derivatives [17].

The aromatic fraction spans 110–140 ppm (unsubstituted and C‐substituted phenyl carbons) and 140–160 ppm (O‐substituted aromatic carbons in polyphenolic molecules and lignin derivatives with C3 and C5 bound to methoxyl carbons) [17]. Finally, the peak at 174 ppm is linked to carbonyl carbons in aliphatic acids, amides, pectins, or secondary oxidation products [26]. The evaluation of the structural index pointed out a balanced distribution of polar and apolar components, with contributions from aliphatic and aromatic compounds (Table 2). Furthermore, the LigR highlights the predominant presence of lignin fragments [16, 17].

#### 3.1.4. Offline TMAH–Pyr–GC–MS

Methyl ethers and esters of alkyl and aryl compounds, derived from plants and microbes, were identified as the main thermochemolysis products of the HS extract (Table 3). These were categorized by origin: lignin units (Lig), methyl esters of fatty acids (FAME), nitrogenous compounds (N), alicyclic compounds (e.g., sterols), and derivatives of carbohydrate polysaccharides (Carb). The relative content analysis confirmed significant lignin incorporation in the humic materials (Table 3). The lower carbohydrate content compared to NMR data aligns with prior studies highlighting the inefficiency of thermochemolysis in detecting polyhydroxyl molecules in complex systems [17, 18].

**Table 3:** Relative yield (%) of main thermochemolysis products released from humic substances (HS) extracted from a coffee grounds (COF) composted residue.

Thermochemolysis products	HS‐COF
Lignin	58.6
Lig G6/G4	7.5
Lig S6/S4	22.6
Aromatic (non‐lignin)	8.9
N derivatives	19.4
FAME	11.4
Sterols	1.4
Carb	1.2

*Note*: The coefficients of variation were invariably smaller than 5%.

Lignin monomers were classified using standard nomenclature: P = *p*‐hydroxyphenyl, G = guaiacol (3‐methoxy, 4‐hydroxyphenyl), and S = syringyl (3,5‐dimethoxy, 4‐hydroxyphenyl) [27]. The predominant monomers included oxidized derivatives of both di‐ and tri‐methoxyphenylpropane, such as benzaldehydes (G4, S4), acetophenones (G5, S5), and benzoic acids (G6, S6). Other tracked components were the cis and trans isomers of 1‐(3,4‐dimethoxyphenyl)‐2‐methoxyethylene (G7, G8), 1‐(3,4, 5‐trimethoxyphenyl)‐2‐methoxyethylene (S7, S8), and the enantiomers of 1‐(3,4‐dimethoxyphenyl)‐1,2,3‐trimethoxypropane (G14 and G15) and 1‐(3,4,5‐trimethoxyphenyl)‐1,2,3‐trimethoxypropane (S14 and S15) [17, 18]. Additional aromatic compounds, including phenols, methyl‐phenols, and alkyl‐benzenes, were identified as originating from lignin or polyphenols or as by‐products of thermochemolysis [19]. The ratio of intact (e.g., G14/14, S14/15) to oxidized lignin forms (G4/6, S4/6) serves as a structural indicator of degradation stages [17]. The benzoic *vs* aldehyde ratios reflect preferential solubilization of depolymerized plant tissues in the humic extract.

#### 3.1.5. Antioxidant Capacities of HS

HSs exhibit strong antioxidant activity due to their complex molecular structures enriched with functional groups like phenolics and quinones. These groups can donate hydrogen atoms or electrons to neutralize free radicals effectively [28]. The antioxidant mechanisms of HS include free radical scavenging, metal ion chelation (e.g., iron and copper) [29], and redox stabilization of reactive species [30].

These properties make HS promising for biomedical applications, particularly in preventing oxidative damage to human cells, thereby promoting health and longevity [18, 19, 26].

In our study, HS demonstrated significant antioxidant capacity in ABTS and DPPH assays, with percentage inhibitions of 68% and TEAC values of 367.8 and 276.7 mmol Trolox equivalents per kg, respectively (Figure S1). The high antioxidant activity strongly correlated with the TPC of 256.67 mmol GAEs per kg, as measured by the Folin‐Ciocalteu test. Based on both NMR and thermochemolysis results (Tables 2 and 3), lignin fragments (e.g., ClA) are the primary compounds responsible for the antioxidant properties of HS [18, 26].

### 3.2. Biological Activity of HS‐COF

#### 3.2.1. HS‐COF Impacts Human Cancer Cell Viability: HT‐29 *versus* PANC‐1

To investigate the cytotoxic properties of HS‐COF on human cancer cells, the MTT assay was performed on human colorectal and pancreatic carcinoma cell lines (HT‐29 and PANC‐1 cells, respectively). As illustrated in Figure 3, HT‐29 cells exhibited a greater susceptibility to the HS than PANC‐1 cells. In detail, HS‐COF reduced cell viability of HT‐29 cells in a dose and time‐dependent manner (IC_50_ = 422.50 μg/mL at 24 h and 349.5 μg/mL at 72 h; Table S2). Interestingly, it showed a slight inhibitory effect on HT‐29 cells already at the lowest tested concentrations (1–5 μg/mL; Figure 3A), even though cell viability remained in the subtoxic range (>70%). On the contrary, HS‐COF showed a mild inhibitory effect on PANC‐1 cells starting from higher concentrations (Figure 3B), and surprisingly, the cytotoxic effect did not appear to be time‐ and dose‐dependent (IC_50_ = 655.0 μg/mL at 24 h and 931.6 μg/mL at 72 h; Table S2).

Figure 3HS‐COF significantly reduces CRC cell viability. Cell viability of HT‐29 (A) and PANC‐1 (B) cell lines was measured by the MTT assay, following treatment with HS‐COF at different concentrations (1 to 500 µg/mL) for 24 and 72 h. Data are represented as averages ± SD of at least two independent experiments, each performed in triplicate. One‐way ANOVA followed by Bonferroni post‐hoc correction was used to compare each treatment with untreated cells (CTR). ∗∗∗∗, *p* < 0.0001.

**Figure:**
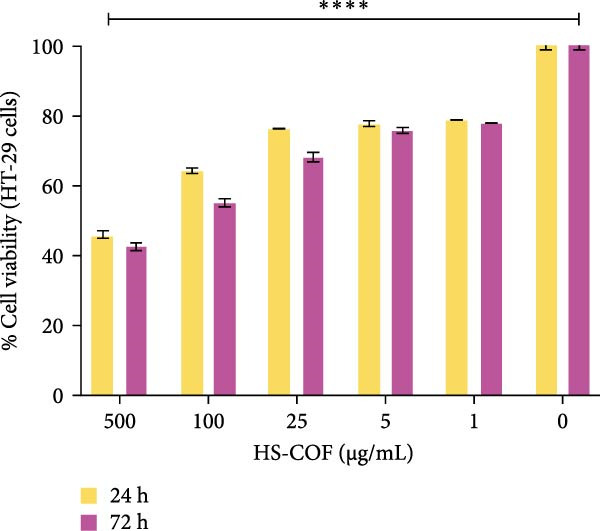
(A)

**Figure:**
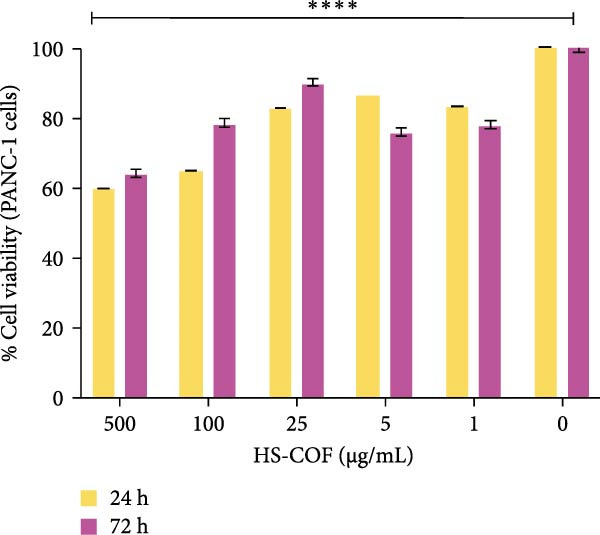
(B)

To better understand the divergent responses of HT‐29 and PANC‐1 cells to HS‐COF treatment, it is essential to consider their distinct molecular patterns. HT‐29 cells display pronounced genomic instability, characterized by numerous genetic and epigenetic alterations [31], including somatic mutations in *APC* (codons 853 and 1555) [32] and *Tp53* (R273H) genes [31]. Although these mutations have different functional outcomes—loss of function in *APC* and gain of function in *Tp53*–both contribute to abnormal cell proliferation through aberrant activation of the Wnt/*β*‐catenin signaling pathway and alteration of the NF‐*κ*B transcriptional activity [31–34]. Notably, a negative crosstalk between these two pathways has been described in CRC cells. In particular, overexpression of *β*‐catenin has been found to suppress NF‐*κ*B transcriptional activity, leading to reduced expression of its downstream target genes, including key pro‐apoptotic mediators such as FAS ligand and TNF‐*α* [35, 36]. As a result, the coexistence of *Tp53* ^*R273H*^ and *APC* ^*853/1555*^ mutations primarily leads to repression of apoptotic signaling in HT‐29, promoting cell survival and tumor progression. Importantly, since both mutations impair interconnected pathways that play critical roles in apoptosis regulation, HT‐29 cells may retain a degree of vulnerability to cytotoxic agents capable of restoring the apoptotic signaling, such as the HS‐COF.

In contrast, PANC‐1 cells harbor oncogenic mutations in *KRAS* (G12D) and *Tp53* (R273H) genes, along with homozygous deletion of *CDKN2A/p16*, a combination of alterations that drive unchecked proliferation [37, 38]. Specifically, constitutive activation of *KRAS* stimulates both the RAF/MAPK and the PI3K/Akt/mTOR pathways, promoting cell growth, proliferation, metastasis and COX‐2 transcription increase, which results in prostaglandin E_2_ (PGE_2_) release and resistance to cytotoxic therapies [38, 39]. In addition, inactivation of *Tp53* and *CDKN2A/p16* impairs the G1/S cell cycle checkpoint control, contributing to genomic instability and cancer progression [38]. These genetic traits are further reinforced by the biological nature of the cell line—a primary tumor‐derived cell line with most of cells in the mitotic phase—as well as by impaired death receptor signaling and upregulation of antiapoptotic mediators, which collectively confer marked resistance to proapoptotic stimuli and contribute to the well‐known aggressive nature of pancreatic cancer [40].

Based on these findings, we excluded PANC‐1 cells from further analyses and focused on HT‐29 cells, concentrating our attention on the potential application of HS‐COF against CRC disease.

#### 3.2.2. HS‐COF Doesn’t Impact Human Noncancer Cell Viability

**Figure 4:**
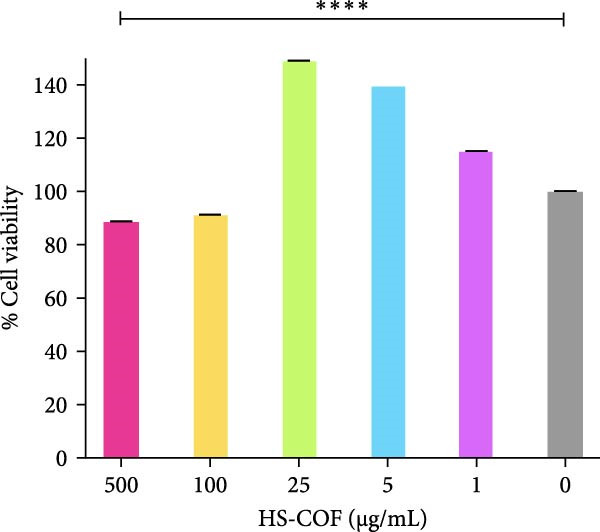
HS‐COF does not exhibit cytotoxic effects on non‐cancerous colonic cells. Cell viability of normal human colonic cells (CCD‐18Co) was measured by the MTT assay. Cells were treated with HS‐COF at different concentrations (1 to 500 µg/mL) for 24 h. Data are represented as averages ± SD of at least two independent experiments, each performed in triplicate. One‐way ANOVA followed by Bonferroni post‐hoc correction was used to compare each treatment with untreated cells (CTR). ∗∗∗∗, *p* < 0.0001.

To assess whether HS‐COF could selectively induce toxicity in cancerous cells, we also performed the MTT assay on noncancer cells. The normal human colonic cell line CCD‐18Co was selected as the counterpart control of the CRC cells HT‐29. In response to the treatment with the highest concentrations of HS‐COF (500 and 100 μg/mL), CCD‐18Co cells exhibited growth inhibition lower than 20% (10.9% and 8.4%, respectively), compared to untreated cells (Figure 4). This result suggests that HS‐COF does not affect the viability of normal colonic cells. Intriguingly, upon treatment with low concentrations, the CCD‐18Co cells increased their viability compared to control cells and, notably, even though cell viability was higher than control cells, it decreased in a dose‐dependent manner (Figure 4). This indicates that HS‐COF has bimodal concentration‐dependent effects on fibroblast‐like cell growth. Low concentrations (1–25 μg/mL) stimulate cell growth, while high concentrations (> 100 μg/mL) decrease cell growth, even though without resulting in cytotoxic.

#### 3.2.3. HS‐COF Induces a Nonreversible Cytotoxicity in HT‐29 Cells

**Figure 5:**
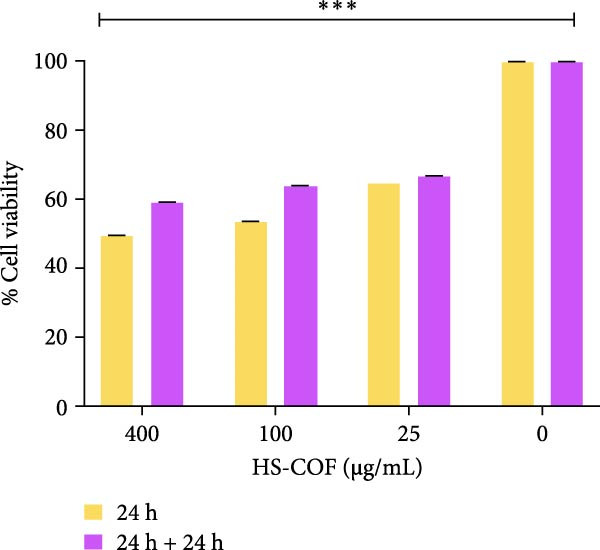
HS‐COF irreversibly reduces CRC cell viability. The HT‐29 cell line was treated with HS‐COF at different concentrations (400; 100 and 25 μg/mL) for 24 h or 24 h + 24 h of recovery and cell viability was measured by the MTT assay. Data are represented as averages ± SD of at least two independent experiments, each performed in triplicate. One‐way ANOVA followed by Bonferroni post‐hoc correction was used to compare each treatment with untreated cells (control). ∗∗∗, *p* < 0.001.

To investigate whether HT‐29 cells recover the viability after HS‐COF treatment, we incubated HT‐29 cells with both the IC_50_ concentration (400 μg/mL) and two other concentrations (100 and 25 μg/mL) of HS‐COF for 24 h. After the treatment, cells were washed and incubated with fresh medium for a further 24 h. Results showed no changes in cell viability when cells were treated with HS‐COF 25 μg/mL, after 24 h of recovery (Figure 5). In contrast, only a limited recovery was observed in cells treated with the IC_50_ concentration or with the concentration of 100 μg/mL (10% and 11%, respectively) (Figure 5). However, cell viability was always lower than 70%.

These results indicate that HS‐COF induces a cancer cell death program which inhibits cell recovery following cytotoxic‐drug removal.

#### 3.2.4. Chlorogenic Acid: The HS‐COF Bioactive Phenolic Compound

ClA is a polyphenol compound, exhibiting anticancer activities [41, 42]. As the most abundant phenolic compound derived from lignin residues—the major chemical components of HS‐COF (Tables 2 and 3)—the content of ClA was evaluated. Chemical analyses revealed that 500 μg/mL of HS‐COF contains a two‐fold increased concentration of ClA compared to caffeic acid (CA) (243.45 μM ± 0.06 and 176.32 μM ± 0.01 mM; respectively) (Figure S2). As a consequence, the effect of ClA on HT‐29 cell viability was investigated by performing the MTT assay. Results showed that ClA reduced cell viability of 68% and 73.3%, after 24 and 72 h of treatment, respectively, at the concentration contained in the IC_50_ = 400 μg/mL of HS‐COF (200 μM) (Figure S3). Based on these findings, it could be reasonable to hypothesize ClA as the primary compound responsible for the biological activity of HS‐COF.

#### 3.2.5. HS‐COF Inhibits HT‐29 Cell Proliferation and Migration

**Figure 6:**
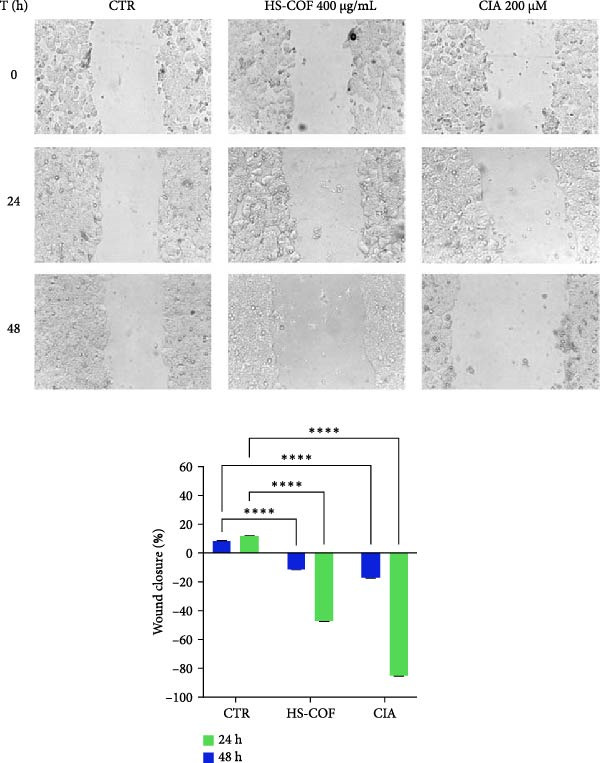
HS‐COF decreases the CRC cell migration capabilities. Cell migration of HT‐29 cells treated with HS‐COF at IC_50_ value (400 μg/mL) or ClA 200 μM for 48 h was measured by a wound healing assay. Data are expressed as averages ± SD of at least two independent experiments, each performed in triplicate and represent the % of wound closure after 24 and 48 h of treatment. Two‐way ANOVA followed by Turkey post‐hoc correction was used to compare each treatment with untreated cells (CTR). ∗∗∗∗, *p* < 0.0001.

To grow and form metastasis, cancer cells use wound healing pathways [43]. Therefore, to ascertain whether HS‐COF could suppress HT‐29 cell migration, a wound healing assay was performed. Our data clearly show that cells treated with the IC_50_ value of HS‐COF (400 μg/mL) significantly inhibit cell migration, compared to untreated cells. Interestingly, HS‐COF not only impedes wound closure but also increases the wound size, although to a lesser extent compared to ClA (Figure 6).

Furthermore, consistent with these findings, HS‐COF was observed to inhibit cell proliferation and suppress the ability of colonic cancer cells to form clonal populations (Table 4).

**Table 4:** Clonal expansion of HT‐29 cells following HS‐COF or ClA treatment.

Treatment	N. of colonies	N. of colonies compared to control (%)	Reduction of colonies compared to control (%)
Control	44.5 ± 0.7	100	0
HS‐COF 400 μg/mL	16.5 ± 0.7	37	63
ClA 200 μM	13.5 ± 2.12	13.5	86.5

*Note*: N. of colonies is expressed as the means ± SD of two independent experiments, each performed in duplicate.

Overall, these results strongly suggest the potential of HS‐COF as a candidate antitumor agent, inducing HT‐29 cell death.

#### 3.2.6. HS‐COF Induces HT‐29 Cell Apoptosis via Extrinsic Pathway

To investigate whether the HS‐COF treatment induces a programed cell death in HT‐29 cells, a DNA ladder assay was performed to detect DNA fragmentation, a key hallmark of apoptosis [44]. The results showed a significant DNA fragmentation in cells treated with HS‐COF, compared to both untreated cells and cells treated with ClA (Figure 7A). Additionally, we also observed an increase in intracellular Ca^++^ levels, critical regulator of the apoptotic signaling pathway [45, 46]. Specifically, cells treated with ClA exhibited higher levels of cytosolic Ca^++^ than those treated with HS‐COF (*p* < 0.05; Figure 7B). This observation aligns with qRT‐PCR results, which demonstrated increased expression of the *Tp53* gene in ClA‐treated cells compared to those treated with HS‐COF (Figure 7C). Conversely, decreased levels of *TNF-α* and *FAS ligand* (*FASL*) gene expression were detected in cells treated with ClA, compared to those treated with HS‐COF (Figure 7D, E). These findings suggest that although both ClA and HS‐COF induce apoptosis in HT‐29 cells, as confirmed by increased expression levels of CASP3 and CASP9 in both HS‐COF and ClA‐treated cells (Figure 7F), they activate different downstream pathways. Consistent with the literature, ClA was found to activate the intrinsic apoptotic pathway [47–49], while HS‐COF emerges as a suppressor of CRC cell viability primarily via the extrinsic signaling pathway. As shown in Figure 7F, in fact, cells treated with HS‐COF displayed reduced CASP9 activity with respect to CASP3, in opposition to cells treated with ClA, which displayed an elevation of CASP9 activity with respect to CASP3. Interestingly, cells treated with HS‐COF displayed higher expression levels of the two CASPs than those treated with ClA, validating the results from the ladder assay (Figure 7A) and suggesting HS‐COF as a valuable apoptotic agent targeting the TNF‐*α* signaling.

Figure 7HS‐COF restores the HT‐29 cell apoptotic pathway. (A) Apoptotic DNA ladder in 2% agarose electrophoresis gel. Lane M: Molecular weight marker (1 kb); Lane a: untreated cells; Lane b: cells treated with DMSO for 24 h; Lane c: cells treated with HS‐COF 400 μg/mL for 24 h; Lane d: cells treated with ClA 200 μM for 24 h. (B) Intracellular Ca^++^ levels of HT‐29 cells treated or not with HS‐COF 400 μg/mL or ClA 200 μM for 24 h. (C–E) mRNA expression levels of *Tp53* (C), *FASL* (D) and *TNF-α* (E) genes detected by quantitative PCR (qRT‐PCR) in HT‐29 cells treated or not with HS‐COF 400 μg/mL or ClA 200 μM for 24 h. Each sample was normalized to *GAPDH* as housekeeping reference gene. (F) Expression levels of CASP3 and CASP9 detected by ELISA in HT‐29 cells treated or not with HS‐COF 400 μg/mL or ClA 200 μM for 24 h. Graphs represent averages ± SD of at least two independent experiments, each performed in triplicate. Ordinary One‐way ANOVA followed by Turkey post‐hoc correction was used to perform the following comparisons: HS‐COF *vs* ClA and HS‐COF or ClA *vs* untreated cells (CTR). ∗∗, *p* < 0.01; ∗∗∗, *p* < 0.001; ∗∗∗∗, *p* < 0.0001.

**Figure:**
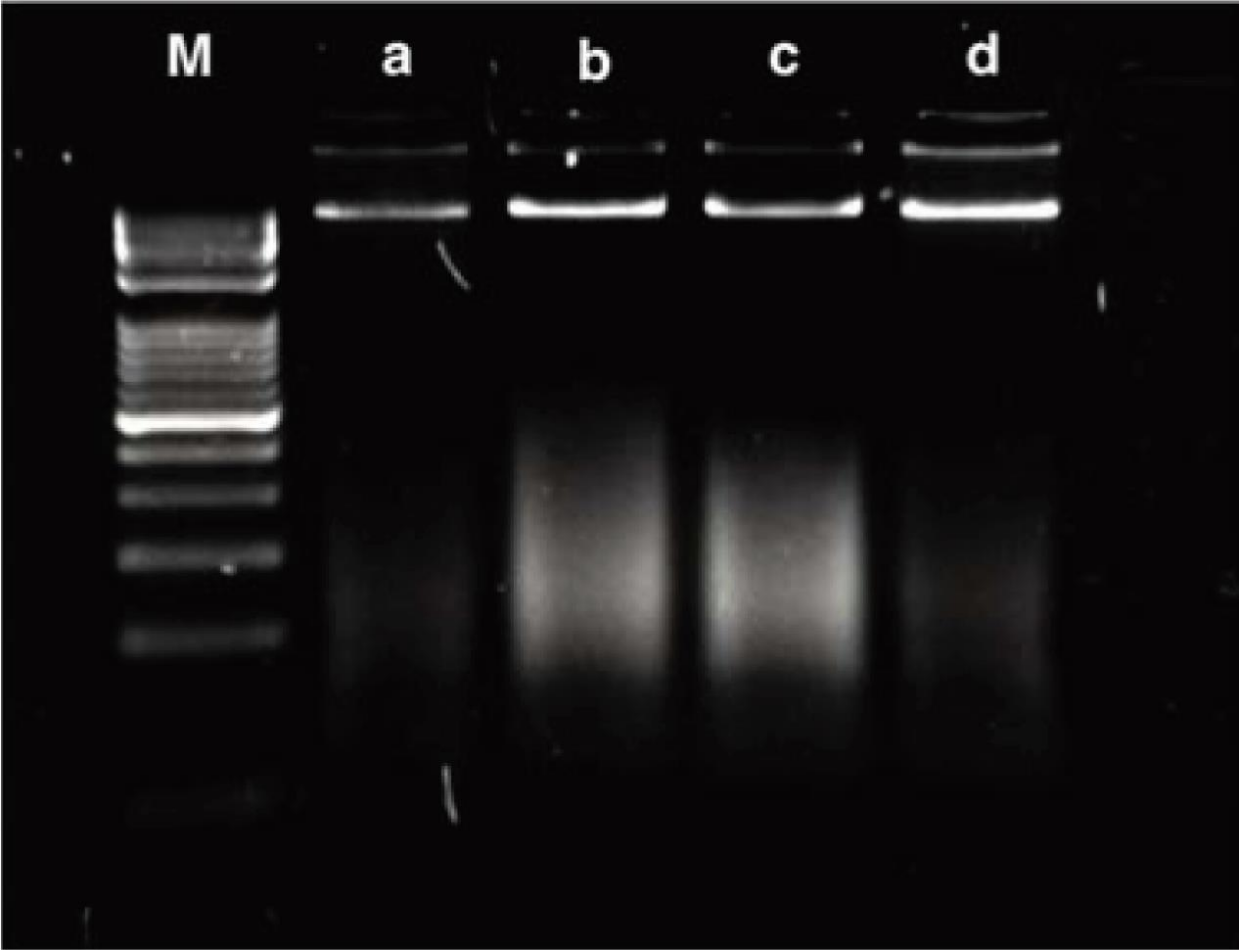
(A)

**Figure:**
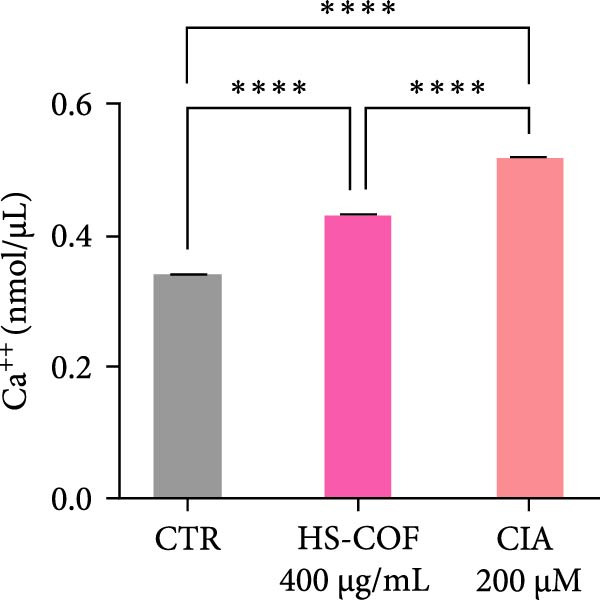
(B)

**Figure:**
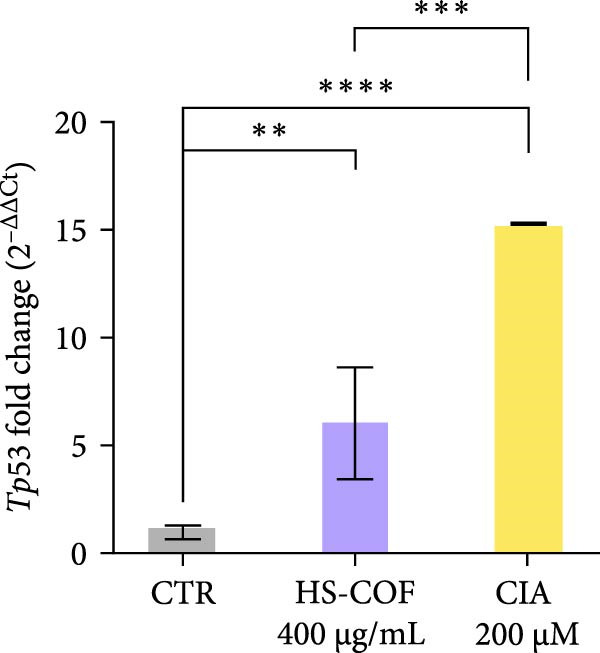
(C)

**Figure:**
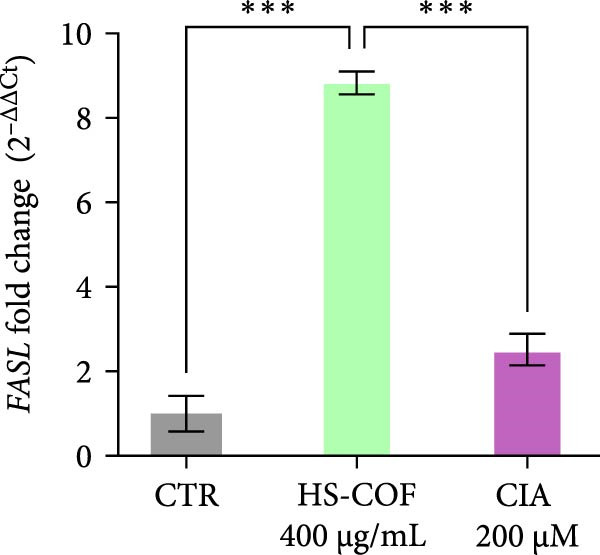
(D)

**Figure:**
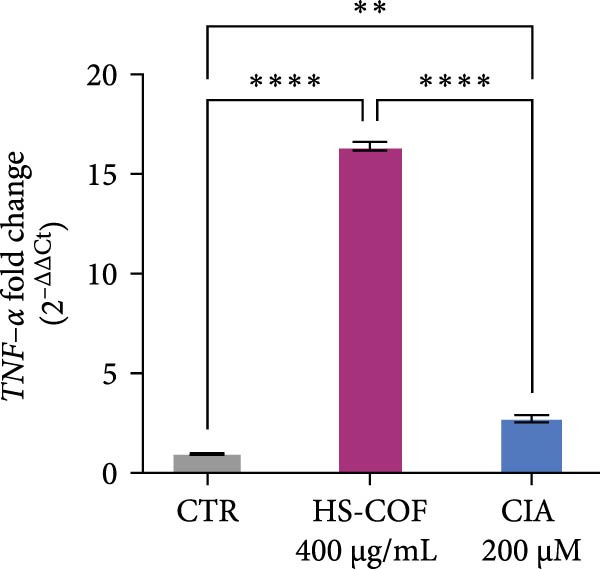
(E)

**Figure:**
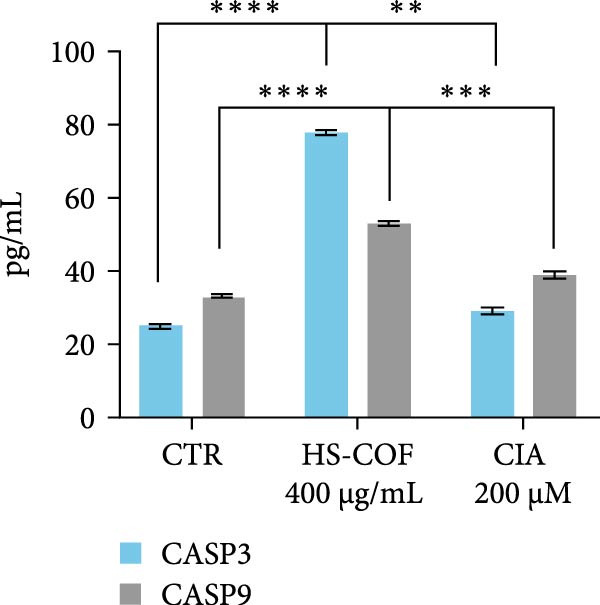
(F)

**Figure 8:**
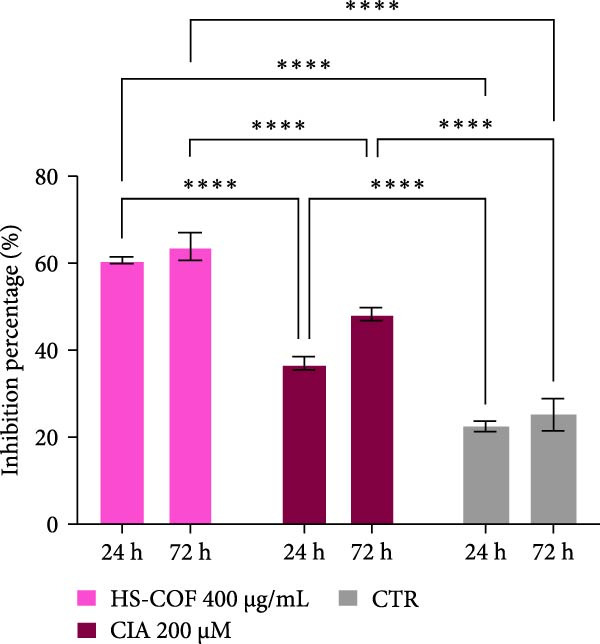
HS‐COF significantly reduces the intracellular concentration of ROS. Antioxidant status of HT‐29 cells treated with HS‐COF at IC_50_ value (400 μg/mL) or ClA 200 μM for 24 and 72 h. Data are represented as averages ± SD of at least two independent experiments, each performed in triplicate and represent the ABTS scavenging ability of HT‐29 cell lysate after 24 and 72 h of treatment. Statistical analysis was performed using two‐way ANOVA followed by Turkey post‐hoc correction for multiple comparisons. ∗∗∗∗, *p* < 0.0001.

Finally, we analyzed the potential of HS‐COF to scavenge ROS. Both ClA and HS‐COF exhibited significant antioxidant activity (Figure 8). Notably, HS‐COF was more effective at inhibiting free radicals than ClA (Figure 8), due to the presence of numerous phenolic groups (Figure 2, Tables 2 and 3), which can neutralize reactive species and chelate metal ions, thus preventing oxidative damage.

Taken together, these results suggest HS‐COF as a promising candidate for CRC treatment, capable of inhibiting cancer cell viability while concurrently controlling oxidative stress, a common side effect of conventional antitumoral drugs causing serious damage to healthy cells.

## 4. Discussion

Malignant cells acquire abnormal growth and survival characteristics [50]. They exhibit an uncontrolled proliferation, due to genetic aberrations that enable them to escape physiological mechanisms governing cell growth and division, including the apoptotic cell death program [51].

In this scenario, the preferred way to limit tumor progression is to control cancer cell proliferation using apoptosis as a therapeutic target. However, this approach has a serious limitation. Most of conventional and successful cytotoxic drugs cannot distinguish between malignant and healthy proliferating cells.

The goal of the present study was to investigate the capacity of HS‐COF to inhibit CRC cell proliferation and migration while limiting adverse effects on healthy cells. We first tested the cytotoxic effect of HS‐COF on HT‐29 cells. Our results showed a significant and nonreversible reduction of cell viability in a dose‐ and time‐dependent manner (Figures 3 and 5). Notably, HS‐COF did not exert cytotoxic effects on normal human colon cells (CCD‐18Co) (Figure 4).

CCD‐18Co cells are stromal cells localized in the lamina propria of the colon mucosa, where they play a central role in maintaining tissue homeostasis by mediating extracellular matrix (ECM) remodeling, wound repair, and immune responses [52]. They display a specific gene‐expression pattern that differentiates them from other cells and fibroblasts of distinct anatomical sites [53, 54]. This profile includes growth and differentiation factors (PTPRM, MAFB), migration guidance molecules (SEMA3C, PLEXIN C1, SLIT3, F‐spondin), ECM components (COL6A1), and members of the Wnt signaling pathway (WISP2 and DAAM2) [54]. Furthermore, in response to specific stimuli, CCD‐18Co fibroblasts upregulate markers such as vimentin (VIM) and TGF‐β1, which cooperate to sustain fibroblast proliferation [55].

In line with the existing literature showing a dual, dose‐ and time‐dependent effect of waste‐derived biomaterials on fibroblast growth [56], HS‐COF significantly increased CCD‐18Co viability at low concentrations and decreased it at higher doses. This bimodal behavior may be attributable to the high phenolic content of HS‐COF. At low concentrations, phenolic compounds may support fibroblast homeostasis by reducing basal oxidative stress and enhancing constitutive proliferation pathways. On the contrary, at higher concentrations, an excessive phenolic content may perturb redox balance and shift cells toward stress‐induced growth inhibition [56]. Importantly, even at the highest tested concentration (500 μg/mL), HS‐COF did not elicit any cytotoxic effect in fibroblasts (Figure 4). In contrast, in HT‐29 cells, which rely on oncogenic signaling pathways and exhibit elevated ROS levels [57], HS‐COF exacerbates intracellular stress and activates death signaling, even at low doses. This highlights selective toxicity towards CRC cells and potential safety for normal tissue.

These findings are strongly supported by the potent antioxidant properties of HS‐COF. As illustrated in Figure 8, HS‐COF showed a two‐fold higher antioxidant effect than ClA alone (60% *vs* 36%; respectively), likely attributable to the synergistic action of ClA and other phenolic compounds—especially lignin derivatives–contained in the HS, which are known for their ROS‐scavenging capabilities.

The molecular characterization of humic materials, together with the uncovered bioactive effects, outlines the occurrence of structural activity relationships. The combination of hydrophilic/hydrophobic domains made up by the association of linear and rigid aromatic components draws a conformational feature that leverages a feasible role as a composite carrier whose surface tension may boost an adequate and intimate interaction with cell structures [28, 58]. Upon interaction with cell membranes, the disassembling of depolymerized pliable colloidal particles is conducive to the unfolding of bioactive and antioxidant metabolites trackable as soluble phenolic and lignin units retained in humic extracts [19, 26].

Based on these considerations, the incorporation of HS‐COF into common cancer treatment regimens could represent a promising approach to control tumor progression and—at the same time—protect normal cells from oxidative damage, ensuring redox balance. Remarkably, this combination not only might improve the overall tolerability of conventional treatments but might also contribute to enhancing their efficacy by promoting cancer cell death.

HS‐COF was found to stimulate cancer cell apoptosis primarily through the extrinsic pathway. A clear demonstration was provided by increased transcription levels of *TNF-α* and *FASL* genes in cells treated with HS‐COF, compared to both untreated and ClA‐treated cells (Figure 7D, E). TNF‐*α* and FASL are death signals and members of the TNF superfamily [59]. Both the proteins are established to initiate the extrinsic signaling pathway of apoptosis upon interaction with their respective death receptors, TNF receptor 1 (TNFR1) and FAS (CD95/Apo1) that, following recruitment of adaptor proteins—such as TNFR1‐associated death domain (TRADD) and FAS receptor‐associated death domain (FADD), respectively–allow the formation of the intracellular death‐inducing–signaling complex (DISC). This multiprotein complex favors the activation of the caspase initiator CASP8 and, consequently, of executer caspases such as CASP3 and CASP7 [60].

Interestingly, recent evidence also indicates the compelling activity of TNF‐*α* to engage the intrinsic mitochondrial pathway of apoptosis via CASP8‐mediated Bid cleavage and subsequent mitochondrial outer membrane permeabilization, suggesting a clear interplay between the extrinsic and intrinsic apoptotic pathways [61, 62].

Members of the Bcl family are key mediators of intrinsic apoptosis. They are grouped into proapoptotic (e.g., Bax, Bad, Bak) and anti‐apoptotic (Bcl‐2 and Bcl‐X_L_) proteins, which modulate the death signaling according to their relative expression [61]. Bcl‐2 overexpression leads to apoptosis suppression by sequestering pro‐apoptotic proteins and modulating the intracellular Ca^++^ fluxes [63]. Based on these considerations, the increased intracellular calcium levels observed in HS‐COF‐treated cells compared to untreated cells (Figure 7B), seem to suggest the involvement of mitochondria in the HS‐COF‐induced cell death program. To corroborate this speculation, we investigated the expression levels of the *Tp53* gene encoding for the tumor suppressor protein p53. Known as the “guardian of the genome”, p53 coordinates DNA repair and apoptotic processes. In response to stress conditions and DNA damage, p53 activates pro‐apoptotic genes, while repressing those with anti‐apoptotic properties. These functions culminate in cell cycle arrest and mitochondrial programed cell death, mechanisms that prevent the propagation of cells with severe DNA damage and preserve genome stability to reduce the risk of cancer development [64].

The HT‐29 cell line is established to harbor a missense mutation (R273H) within the DNA‐binding domain of *Tp53* gene, which gains new oncogenic functions. One of the major mechanisms underlying the oncogenic functions of the *Tp53* ^*R273H*^ is the interaction with p63/73 proteins [65, 66]. Such a mechanism inactivates the two proteins and activates the PI3K/Akt signaling pathway, which promotes downregulation of both the Bcl‐2 protein and Bcl‐2‐modifying factor (BMF) and stabilization of the BIM/BCL‐X_L_ complex, thus suppressing the mitochondria‐dependent apoptosis process while fostering invasive and metastatic signaling [66]. Of note, *Tp53* ^*R273H*^ can also impair the apoptotic process through aberrant activation of the NF‐kB activity and Wnt/*β*‐catenin signaling pathway [33, 67].

The Wnt signaling is the key regulator of the intestinal epithelium homeostasis. It mediates the intestinal mucosa renewal by promoting enterocyte apoptosis through the activation of apoptotic mediators, including the receptors TRAIL and FAS, which trigger the extrinsic pathway [68, 69]. In this context, the *APC* gene has a crucial role. Loss‐of‐function mutations in this gene, such as the one occurring in HT‐29 cells (*APC* ^*853/1555*^), lead to *β*‐catenin overexpression and apoptosis repression, likely attributable to the inhibitory effect of *β*‐catenin on the NF‐kB transcriptional activity, which inhibits both TNF‐*α* and FASL production [35].

Given this scenario, it is evident that the coexistence of *TP53* ^*R273H*^ and *APC* ^*853/1555*^ mutations and their cooperating activity establish a strong antiapoptotic background in HT‐29 cells by repressing both intrinsic and extrinsic pathways. Importantly, the higher transcriptional level of *Tp53* in cells treated with HS‐COF compared to the untreated control (Figure 7C), even though to a lesser extent than in cells treated with ClA, indicate that HS‐COF can also restore the mitochondrial apoptotic signaling other than the extrinsic one, thus validating the above‐illustrated results. Further evidence was provided by upregulation of both CASP9 and CASP3 in HS‐COF–treated cells (Figure 7F). While CASP9 is a hallmark of intrinsic apoptosis, CASP3 acts as the major executioner caspase for both pathways. Therefore, the stronger upregulation of CASP3 compared to CASP9 may support the integration of extrinsic and intrinsic signaling in HS‐COF–induced apoptosis. Taken together, these results indicate that HS‐COF may act at multiple levels of apoptotic regulation, re‐establishing death signaling even in the presence of oncogenic mutations that normally suppress apoptosis. However, additional experiments are needed to confirm the interplay between the pathways, clarify the HS‐COF precise mechanism of action, and determine whether its efficacy is maintained at subtoxic doses (e.g., 25 and 100 µg/mL) and remains specific to CRC cells carrying combined pathway‐disrupting mutations, such as those in HT‐29.

## 5. Conclusions

In conclusion, this study proposes HS‐COF as a promising complementary approach for CRC therapy. The selective cytotoxicity and potent antioxidant properties highlight its potential as a safe and bio‐compatible dietary supplement in cancer treatment, especially as an adjuvant to conventional therapies. Moreover, the use of coffee wastes to produce HS‐COF aligns with sustainable and cost‐effective pharmaceutical practices, adding environmental and economic benefits to its therapeutic potential. Nevertheless, further *in vitro* and *in vivo* studies are needed to validate our findings and explore the full therapeutic potential of HS‐COF, alone and in combination with conventional drugs, in an effort to extend its use to other types of cancers other than CRC.

## Conflicts of Interest

The authors declare no conflicts of interest.

## Author Contributions

**Mariavittoria Verrillo**: writing – original draft, writing – review and editing, investigation, methodology, formal analysis, chemical data curation. **Paola Cuomo**: writing – original draft; writing – review and editing, investigation, methodology, formal analysis, biological data curation. **Cristina Pagano**: writing – review and editing, formal analysis, visualization. **Fabrizio Martora**: writing – review and editing, formal analysis, visualization. **Riccardo Spaccini**: writing – review and editing, supervision, methodology, validation. **Rosanna Capparelli**: writing – review and editing, conceptualization, supervision, methodology, data curation, validation. **Salvatore Velotto**: writing – review and editing, formal analysis, visualization. Mariavittoria Verrillo and Paola Cuomo contributed equally to this work and are co‐first authors.

## Funding

No funding was received for this manuscript.

## Supporting Information

Additional supporting information can be found online in the Supporting Information section.

## Supporting information


**Supporting Information** Figure S1: Antioxidant activity of HS‐COF carried out by different spectrophotometric assay ABTS and DDPH. Figure S2: Caffeic acid (A) and ClB (B) determination in HS‐COF. Figure S3: ClA inhibits the HT‐29 cell viability. Cell viability of the HT‐29 cell line was measured by the MTT assay following treatment with ClA at different concentrations (1000 to 50 µg/mL) for 24 h. Data are represented as averages ± SD of at least two independent experiments, each performed in triplicate. One‐way ANOVA followed by Bonferroni post‐hoc correction was used to compare each treatment with untreated cells (control). ∗∗∗∗, *p* < 0.0001. Table S1: List of primers used for qRT‐PCR. Table S2: IC50 values of HS‐COF in HT‐29 or PANC‐1 cell lines at 24 or 72 h of treatment, determined by linear regression method.

## Data Availability

The data that support the findings of this study are available from the corresponding author upon reasonable request.
