# Supplementary material for: Coffee Wastes: A Sustainable Source of Natural Compounds Suppressing Colorectal Cancer Cell Viability
Source: Oxid Med Cell Longev. 2025 Dec 28;2025:8034350. doi: 10.1155/omcl/8034350 (PMC12767483; doi:10.1155/omcl/8034350)
Supplement: Supplementary file 1 — Supporting Information Figure S1: Antioxidant activity of HS‐COF carried out by different spectrophotometric assay ABTS and DDPH. Figure S2: Caffeic acid (A) and ClB (B) determination in HS‐COF. Figure S3: ClA inhibits the HT‐29 cell viability. Cell viability of the HT‐29 cell line was measured by the MTT assay following treatment with ClA at different concentrations (1000 to 50 µg/mL) for 24 h. Data are represented as averages ± SD of at least two independent experiments, each performed in triplicate. One‐way ANOVA followed by Bonferroni post‐hoc correction was used to compare each treatment with untreated cells (control). ∗∗∗∗, p < 0.0001. Table S1: List of primers used for qRT‐PCR. Table S2: IC50 values of HS‐COF in HT‐29 or PANC‐1 cell lines at 24 or 72 h of treatment, determined by linear regression method. [file OMCL-2025-8034350-s001.docx]

**Supplementary Material**

**Coffee Wastes: A Sustainable Source of Natural Compounds Suppressing Colorectal Cancer Cell Viability**

Mariavittoria Verrillo, Paola Cuomo, Cristina Pagano, Fabrizio Martora, Riccardo Spaccini, Rosanna Capparelli, Salvatore Velotto.

### TABLE OF CONTENTS

### *Supplementary Figures 1-3 Page 1-2*

### Supplementary Figure S1 page 1

### Supplementary Figure S2 page 1

### Supplementary Figure S3 page 2

### *Supplementary Tables 1-2* *Page 3*

### Supplementary Table S1 page 3

### Supplementary Table S2 page 3

###

### SUPPLEMENTARY FIGURES:

###
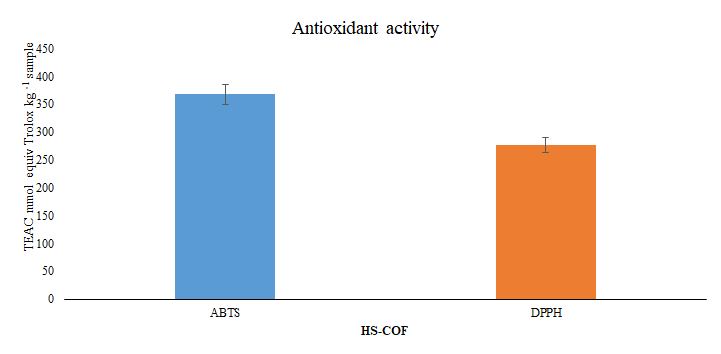


### Supplementary Figure S1

###
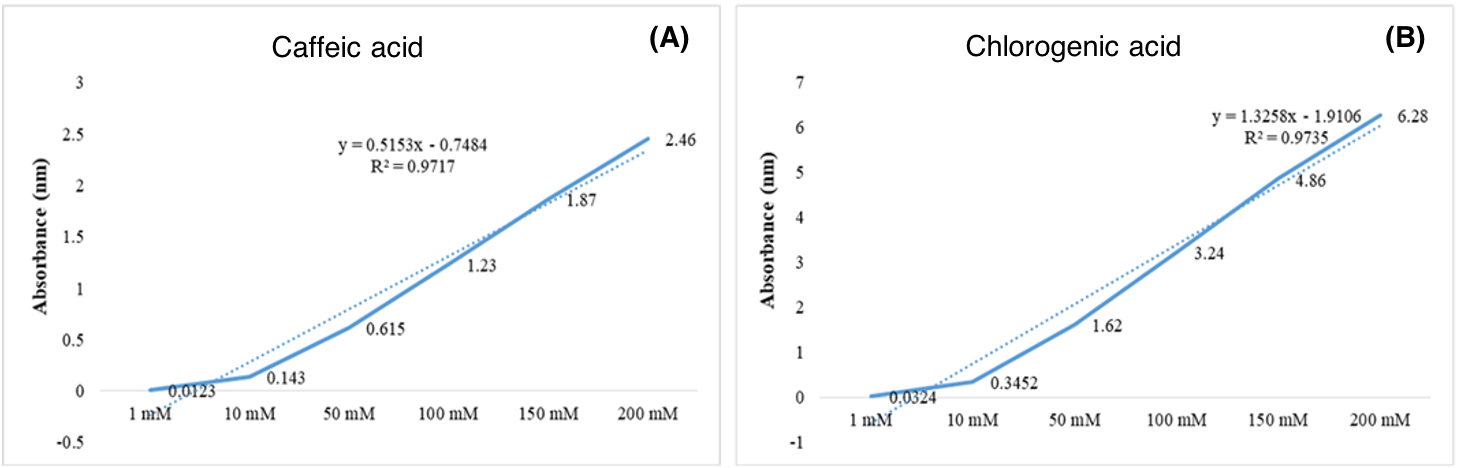


### Supplementary Figure S2

###
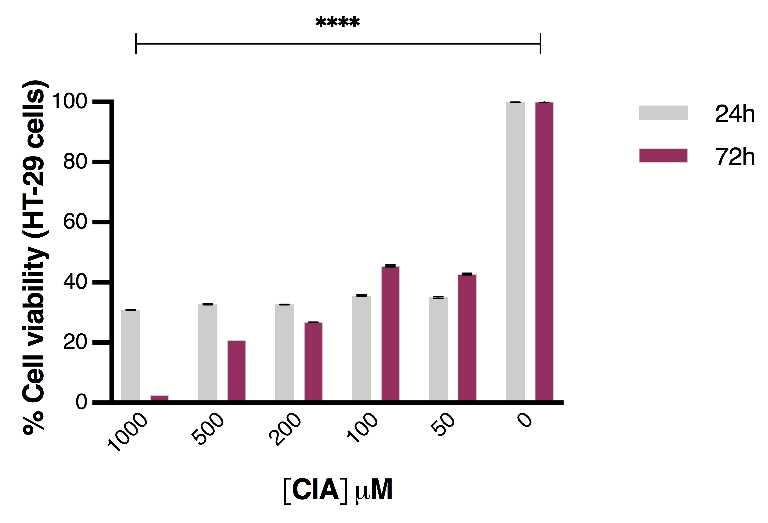


**Supplementary Figure S3**

### SUPPLEMENTARY TABLES:

### Supplementary Table S1

| GENE ID | FORWARD SEQUENCE | REVERSE SEQUENCE |
| --- | --- | --- |
| *GAPDH* | GTCTCCTCTGACTTCAACAGCG | ACCACCCTGTTGCTGTAGCCAA |
| *FAS* | GGTTCTGGTTGCCTTGGTAGGA | CTGTGTGCATCTGGCTGGTAGA |
| *TNF-α* | CTCTTCTGCCTGCTGCACTTTG | ATGGGCTACAGGCTTGTCACTC |
| *Tp53* | CCTCAGCATCTTATCCGAGTGG | TGGATGGTGGTACAGTCAGAGC |

Primers were used according to the following PCR program: Activation: 50 °C for 2 min; Stage 2: pre-soak: 95 °C for 10 min; Stage 3: Denaturation: 95 °C for 15 sec, Annealing: 60°C for 1 min; Stage 4: Melting curve: 95°C for 15 sec, 60°C for 15 sec, 95°C for 15 sec.

**Supplementary Table S2**

| **Cell line** | **24 h**  **Equation R^2^ value IC_50_**  **(µg/mL)** | | | **72h**  **Equation R^2^ value IC_50_**  **(µg/mL)** | | |
| --- | --- | --- | --- | --- | --- | --- |
| HT-29 | y= –0.0636x+76.89 | 0.94 | 422.79 | y= –0.063x+72.019 | 0.80 | 349.5 |
| PANC-1 | y= –0.0478x+81.79 | 0.70 | 665 | y= –0.0342x+81.86 | 0.62 | 931.6 |
